# Cholestasis: exploring the triangular relationship of gut microbiota-bile acid-cholestasis and the potential probiotic strategies

**DOI:** 10.1080/19490976.2023.2181930

**Published:** 2023-03-02

**Authors:** Leilei Yu, Yaru Liu, Shunhe Wang, Qingsong Zhang, Jianxin Zhao, Hao Zhang, Arjan Narbad, Fengwei Tian, Qixiao Zhai, Wei Chen

**Affiliations:** a State Key Laboratory of Food Science and Technology, Jiangnan University, Wuxi, China; b School of Food Science and Technology, Jiangnan University, Wuxi, China; c International Joint Research Laboratory for Probiotics, Jiangnan University, Wuxi, Jiangsu, China; d National Engineering Research Center for Functional Food, Jiangnan University, Wuxi, China; e Gut Health and Microbiome Institute Strategic Programme, Quadram Institute Bioscience, Norwich, UK

**Keywords:** Cholestasis, enterohepatic circulation, bile acids, gut microbiota, probiotic

## Abstract

Cholestasis is a condition characterized by the abnormal production or excretion of bile, and it can be induced by a variety of causes, the factors of which are extremely complex. Although great progress has been made in understanding cholestasis pathogenesis, the specific mechanisms remain unclear. Therefore, it is important to understand and distinguish cholestasis from different etiologies, which will also provide indispensable theoretical support for the development of corresponding therapeutic drugs. At present, the treatment of cholestasis mainly involves several bile acids (BAs) and their derivatives, most of which are in the clinical stage of development. Multiple lines of evidence indicate that ecological disorders of the gut microbiota are strongly related to the occurrence of cholestasis, in which BAs also play a pivotal role. Recent studies indicate that probiotics seem to have certain effects on cholestasis, but further confirmation from clinical trials is required. This paper reviews the etiology of and therapeutic strategies for cholestasis; summarizes the similarities and differences in inducement, symptoms, and mechanisms of related diseases; and provides information about the latest pharmacological therapies currently available and those under research for cholestasis. We also reviewed the highly intertwined relationship between gut microbiota-BA-cholestasis, revealing the potential role and possible mechanism of probiotics in the treatment of cholestasis.

## 1 Introduction

**Figure 1.**
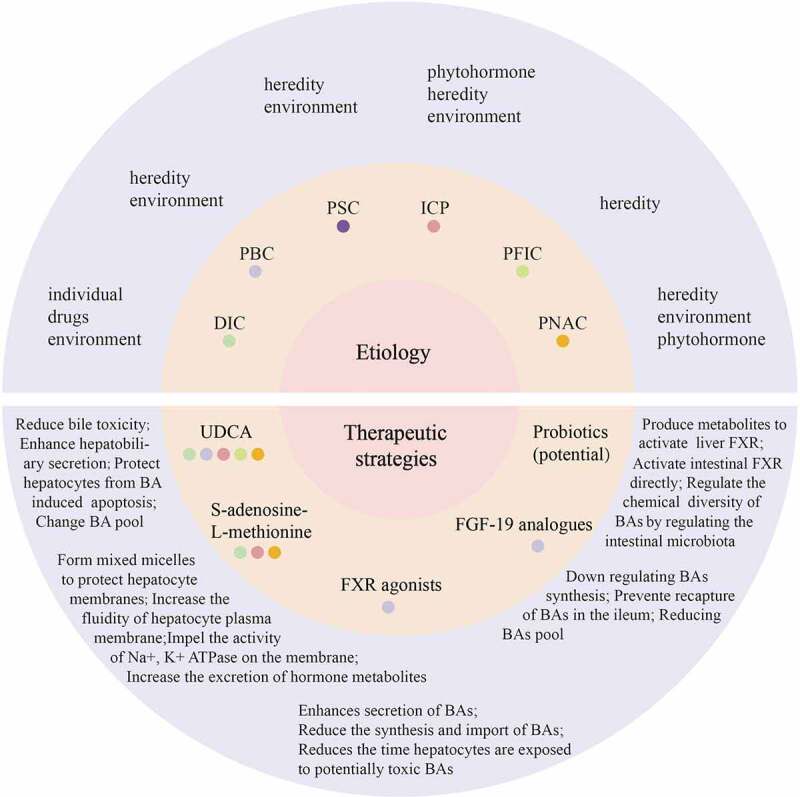
Common pathological factors and therapeutic strategies of cholestasis.

Cholestasis is a pathological state in which bile synthesis, secretion, and excretion are impaired, rendering bile unable to carry out normal liver intestinal circulation. When the formation or excretion of bile is impaired, different consequences may occur. One consequence is that the accumulation of bile exceeds the limits of normal liver cells, resulting in cell injury to the liver parenchyma, toxic damage to the biliary tree, and subsequent obstruction of bile inflow or outflow^1^. The clinical manifestations of cholestasis are fatigue, pruritus, and jaundice, and early biochemical evidence of cholestasis includes elevated serum alkaline phosphatase (ALP) and γ-glutamyl transpeptidase (GGT) levels, conjugated hyperbilirubinemia subsequently develops at a later stage.^2^ Cholestasis can be either acute or chronic (> six months).^3^ Acute factors of cholestasis include biliary obstruction (e.g., choledocholithiasis), cholangitis, drug-induced cholestasis (DIC), parenteral nutrition-associated cholestasis (PNAC), intrahepatic cholestasis of pregnancy (ICP), and alcoholic hepatitis. Chronic factors include DIC, PNAC, primary sclerosing cholangitis (PSC), primary biliary cholangitis (PBC), biliary atresia, and genetic disorders (such as progressive familial intrahepatic cholestasis (PFIC)) (Figure 1).^4^ It is worth noting that these factors can lead to cholestasis, which in turn can exacerbate these diseases further. Some cases of acute cholestasis may progress to chronic manifestations, while chronic cholestasis may present as an acute attack. For instance drugs and total parenteral nutrition are both acute and chronic causes. If left untreated, accompanying liver parenchymal cell toxicity, inflammation, fibrosis progresses, eventually leading to cirrhosis and liver failure.^5^

To date, there have been few effective therapeutic approaches for cholestasis. Only two drugs have been authorized by the FDA to treat the disease. The first is ursodeoxycholic acid (UDCA), a first-line drug for cholestasis. However, approximately 40% of patients are unresponsive to UDCA. Therefore, another drug, obeticholic acid (OCA), is used in patients who are unresponsive or intolerant to UDCA; however, only 50% of patients are responsive.^6,7^ Those who do not respond to UDCA and/or OCA medication are in danger of progressing to biliary cirrhosis, end-stage liver disease, or even death.^8^ Therefore, some novel therapies, including fibroblast growth factor 19 (FGF-19) analogues and nuclear and membrane receptor agonists, have been widely studied.^9^

Probiotics, which are microorganisms with beneficial effects on the human body, play an effective role in various diseases, and their alleviating effect on cholestasis has been reported.^10^ However, the exact mechanisms of action of probiotics at the molecular level remain unknown. The effective role of probiotics is mainly based on the following three levels: (i) direct enzymatic activity and direct interaction with gut microbiota; (ii) interaction with intestinal mucus and epithelium; (iii) signal transmitter, sending signals to the host to reach potential organs, such as the liver.^11,12^ The inducing factors of cholestasis have been described in detail, but their similarities and differences are not fully analyzed in previous reviews.^13,14^ Thus, this paper reviews the etiology of and therapeutic strategies for cholestasis; summarizes the similarities and differences in inducement, symptoms, and mechanisms of related diseases; and provides the latest pharmacological therapies currently available and those under research for cholestasis. Moreover, the drugs for treating cholestasis have been introduced in previous reviews, but the role of the gut microbiota has been ignored.^9,15^ In some recent studies, the beneficial effects of probiotics on cholestasis have also been demonstrated.^16,17^ Therefore, we reviewed the triangular relationship among gut microbiota, BA and cholestasis, revealing the potential role and possible mechanism of probiotics in the treatment of cholestasis.

## 2 Incentive of cholestasis

### 2.1 DIC

DIC is characterized by impaired canalicular bile flow, which leads to the accumulation of harmful bile components in blood and tissues (Table 1). The drug classes most relevant to DIC include anti-infectious, anti-diabetic, anti-inflammatory, psychotropic, cardiovascular agents, and steroids.^46^ However, little is known about risk factors for DIC. The interaction between one or more of these factors, such as age, sex, race, and drug composition, may contribute to the risk of cholestasis.^18^ The true incidence of DIC, a common manifestation of drug-induced liver injury (DILI), cannot be accurately estimated due to the limited number of population-based studies, geographic heterogeneity and underreporting of cases, but in recent years, the incidence of DIC has been increasing year by year as the population ages and the proportion of clinical drug use and combinations increases. In a recently published retrospective study aimed at determining the incidence and causes of DILI in mainland China, DIC was found to be 20.31% of a total of 25,927 cases of confirmed DILI collected in mainland China,^19^ and in a review of DILI case series by Bjornsson et al. the incidence of DIC was reported to be 20% to 40%.^20^ Acute DIC occurs most often, with or without hepatocyte and inflammatory damage, and with vague symptoms such as nausea, malaise, anorexia, and fatigue. Chronic DIC is caused by bile duct or catheter injury, with clinical features such as jaundice, pruritus, melanoderma, and xanthoma.^21^

**Table 1.** Similarities and differences of various etiologies of cholestasis.

	Type	Disease population	Risk factors	Clinical features	Epidemiology	Diagnostic markers	Cholestasis trigger mechanism	References
DIC	Hepatocellular cholestasis, Bile duct cellular cholestasis	More common in the elderly	Individual, Drugs, Environment	Jaundice, pruritus; increased serum ALP, GGT, transaminase	China, 5265/25,927, 20.31% Sweden, 206/784, 26%	Recovery of abnormal liver parameters after drug discontinuation and positive reactions to re-dosing	Three major mechanisms: (i) transporter changes; (ii) hepatocellular changes; (iii) altered bile canalicular dynamics	^3,18–22^
PBC	Bile duct cellular cholestasis	Women in their 5^th^ or 6^th^ decade of life	Heredity, Environment	Fatigue, pruritus, malabsorption syndrome, sicca syndrome, osteoporosis; increased serum ALP, GGT, transaminase	Asia Pacific(7 countries), 118.75/million China, Japan, 191.18 /million Korea, Australia, 39.09 /million	Elevated ALP, Positive AMA, Non-suppurative destructive cholangitis, Interlobular bile duct destruction	the interaction of immune and biliary pathways, which further leads to BEC damage	^23–27^
PSC	Bile duct cellular cholestasis	Men around the age of 40	Heredity, Environment	Pruritus, fatigue, right upper quadrant pain; increased serum ALP, GGT, transaminase	European Union, 5/10,000 Singapore, 0.13/10,000 Japan, 0.095/10,000	Elevated ALP, Elevated GGT, typical multifocal narrowing and segmental dilatation of the bile duct	Intestinal mucosal barrier disorder, bile duct mucosal barrier damage, flora imbalance, immune interaction	^13,28–32^
ICP	Hepatocellular cholestasis	Pregnant woman	Phytohormone, Heredity, Environment	Pruritus; increased serum ALP, GGT, transaminase	Central, Western Europe, North America, Australia, 1% Chile, 5%	Elevated blood glycolic acid ≥10.75 μmol/L or Elevated TBAs ≥ 10 μmol/L	Abnormal BA transporter protein function; The increase of reproductive hormone estrogen and progesterone levels	^14,33–35^
PFIC	Hepatocellular cholestasis	Infants and children	Heredity	Jaundice, pruritus; increased serum BA	1/50,000–100,000, exact prevalence is not know	Genes	Related to mutations in hepatocellular transport-system genes involved in bile formation(FIC1, BESP, MDR3)	^36–40^
PNAC	Hepatocellular cholestasis	Infants are more likely than adults	Heredity, Environment, Phytohormone,	Jaundice; elevated conjugated bilirubin, serum BA, transaminase	Britain, 924/3280, 28.2	two consecutive measurements of direct bilirubin >2 mg/dL	Altered enterohepatic circulation and flora translocation; PN itself or specific components of PN	^41–45^

Three main mechanisms and loci of action are involved in the development of DIC:^3,22^ (i) changes in transporter proteins, that is direct inhibition, internalization, or decreased expression of bile transporter proteins. In particular, the bile salt export pump (BSEP/ABCB11), multidrug resistance-associated protein 2–4 (MRP2-4), sodium/taurocholate cotransporting polypeptide (NTCP/SLC10A1), organic anion transporting polypeptide 1B1/1B3/2B1 (OATP1B1/1B3/2B1), and P-glycoprotein (MDR1) are frequent targets of DIC. Some of these transporters can affect the clearance and excretion of BAs and other bile components, such as cyclosporine A rifampicin, nefazodone, glibenclamide, and troglitazone, which have the capacity to interfere with BSEP activity and may inhibit BSEP, depriving hepatocytes of their primary pathway for BA excretion into the bile ducts, which results in the accumulation of hepatocyte BA to cytotoxic concentrations.^47–49^ Some also take up chemicals in the blood sinusoids, e.g., NTCP transports more than 80% of bound bile acids, and members of the OATP family primarily transport unbound bile acids to the liver, while drugs may inhibit hepatic uptake of BA and disrupt the enterohepatic circulation of bile acids.^50^ Therefore, any drug-related effects on these transport systems may inhibit or degrade transporter proteins, allowing potentially harmful bile acid accumulation or increased uptake of foreign substances by the liver, affecting the liver’s ability to secrete bile, which in turn may cause cholestatic injury. (ii) Hepatocellular changes, including compromised cytoskeletal architecture, disruption of tight junctions, and decreased membrane fluidity. (iii) Changes in bile canalicular dynamics, i.e. dilation or contraction of bile ducts, and spontaneous rhythmic contraction of bile ducts are significant for BAs flow, a process that is related to the frequency of myosin light chain 2 phosphorylation and dephosphorylation. These signals are regulated by the Rho-associated protein kinase (ROCK)/myosin light chain kinase/myosin pathway.^51,52^ These changes can lead to the accumulation of bile and then activate two cellular responses, namely the deterioration response ^53^ (typically the occurrence of mitochondrial damage) and adaptive response^54^ (which strives to activate many nuclear receptors to counteract the accumulation of BAs).

### 2.2 PBC

PBC, previously described as primary biliary cirrhosis, is an autoimmune cholestatic liver disease resulting from immune-mediated biliary tract injury, which can also lead to fibrosis and eventually cirrhosis with associated complications (Table 1). Apart from the assumed essential environmental triggers, the inducement of PBC encompasses immunogenetic risk, epigenetic regulation of biliary epithelia, adaptive and innate immunity, and BA physiology across the gut–liver axis,^23^ which are most commonly recognized in women in their fifth or sixth decade of life.^24^ At least 100,000 people worldwide are diagnosed with PBC each year, and studies show that at least one in every 1,000 women over the age of 40 has PBC.^55^ In the Asia-Pacific region, the prevalence and incidence of PBC appears to be higher than previous reports, with an overall prevalence of 118.75 cases/million (95% CI 49.96–187.55) of PBC found in a meta-analysis that included 18 studies from 7 countries/regions (including Japan, China, New Zealand, Korea, Australia, India and Singapore), with Japan and China had the highest prevalence (191.18 cases/million) and Korea and Australia had the lowest (39.09 cases/million).^25^ Patients with PBC usually exhibit the biochemical features of chronic cholestasis: ALP levels increase to twice the normal upper limit for six months or more; similarly, GGT levels increase to more than five times the normal upper limit. The serological hallmark of PBC is the presence of anti-mitochondrial antibodies (AMA), which is confirmed in approximately 95% of PBC patients.^26^

The interaction of the immune and biliary pathways is the mechanism causing the development of PBC, which further leads to biliary epithelial cells (BECs) damage and eventually to the progression of cholestasis and liver fibrosis.^27^ The simplest way to consider the pathogenesis is that viruses, bacteria, chemicals, etc. can break the body’s self-tolerance to mitochondrial antigens through molecular mimicry, initiating a multilineage immune response against BECs and leading to disease progression.^27^ Persistent innate and adaptive immunity is involved in the further propagation of BECs injury, resulting in chronic inflammation of the bile ducts, which in turn leads to systemic and intrahepatic accumulation of hydrophobic cytotoxic BAs (including CDCA, DCA and lithocholic acid(LCA)).^56^ Therefore, promoting the clearance of liver BAs is the main therapeutic target of PBC.

### 2.3 PSC

PSC is a chronic liver disease characterized by intrahepatic or extrahepatic stenosis, or both (Table 1).^57^ A close association with inflammatory bowel disease (IBD) is a hallmark of this disease, as up to 80% of patients with PSC develop IBD.^13^ Similar to PBC, PSC can develop into fibrosis and cirrhosis, usually lasting for many years, with abnormalities such as bile formation and flow obstruction.^58^ Most patients are male (the ratio of males to females is approximately 2:1) around 40 years of age; however, children and adolescents may also be affected.^28^ PSC conforms to the definition of a rare disease, as its incidence, although varies geographically, it affects less than 200,000 people in the US and about 5 out of every 10,000 inhabitants in the EU with PSC.^13^ There is a geographical gradient between the south and east, with studies in Spain (0.022/10,000), Singapore (0.13/10,000) and Japan (0.095/10,000) reporting an approximately 10-fold reduction in prevalence.^29^ In children, the incidence was 0.23/100,000 per year.^59^ PSC is insidious, and approximately 50% of patients are asymptomatic in its early stages. Symptoms such as pain in the right upper quadrant (20%), pruritus (10%), jaundice (6%), and fatigue (6%) appear as the disease progresses.^60^ Typically, Serum levels of ALP is elevated 2 times the normal upper limit, accompanied by aspartate and alanine aminotransferases. In contrast, 70% of patients had normal serum bilirubin levels at the time of the first episode.^28,61^

However, the pathogenesis of PSC remains unclear. It is now believed that the interaction of the enterohepatic axis plays a role in the pathogenesis of PSC, where intestinal mucosal barrier barriers, dysbiosis and immune interactions are involved in the cholestasis in the pathogenesis of PSC.^62–64^ The pathophysiological basis of bile duct injury is the imbalance of BA homeostasis, damage to the bile duct mucosal barrier and activation of reactive bile duct cells; the presence of reactive T cells, macrophages and neutrophils, predominantly T lymphocytes, around the bile ducts of patients with PSC, and immune disorders are also part of the pathogenesis of PSC.^65^The above factors lead to chronic inflammation and fibrosis of the bile ducts, activation of hepatic stellate cells and myofibroblasts, and interaction with bile duct cells to further aggravate bile duct damage, in which bacteria or bacterial components and products reach the liver through the portal circulation due to the “leaky gut” phenomenon, further causing a systemic inflammatory response in the body and disrupting the tight junctions between BECs.^30,31^ This change exposes biliary cells to BAs, which have a direct toxic effect on biliary cells. Long-term chronic inflammation and toxic damage to the bile ducts can lead to intrahepatic cholestasis, liver fibrosis and even bile duct cancer.

### 2.4 ICP

ICP, also known as obstetric cholestasis, is a liver-specific disorder, affecting approximately 0.5–2.0% of all pregnancies (Table 1).^66^ In general, 80% of ICP cases appear after 30 weeks, but can appear as early as 8 weeks of pregnancy.^67^ Several risk factors for ICP exist, including hormonal, environmental, and genetic factors. The incidence of ICP varies, with a global prevalence of 0.5% to 5.6%, depending on geographical location and ethnicity, and appears to be seasonally related.^68^ The prevalence is less than 1% in Central and Western Europe, North America and Australia, and 1% to 2% in Scandinavia and the Baltic States, but among the Araucanian Indians of Chile and Bolivia, it may be as high as 5% to 15%.^69^ ICP is more common and appears earlier in women with multiple pregnancies.^33^ ICP is often accompanied by pruritus in the third trimester (mainly palms, soles, and limbs), serum BAs, increased liver transaminases, and occasionally bilirubin.^14^ In addition to maternal symptomatology, ICP may be related to an increased incidence of adverse pregnancy outcomes including spontaneous preterm birth, meconium-stained amniotic fluid, fetal distress, fetal asphyxia events, and intrauterine death. .^34,67^

The pathogenesis of this disorder is unclear, however, it can be summarized in two points. (i) Abnormal BA transporter protein function. Some studies have shown that the development of ICP may be associated with the genetic diversity of the expression of hepatobiliary transporters or their nuclear regulators, as well as gene mutations.^70^ Defects in at least seven genes^66^ (ATP8B1 (encoding FIC1), ABCB11 (encoding BSEP), BCB4 (encoding MDR3), ABCC2 (encoding MRP2), NR1H4 (encoding the farnesoid X receptor, FXR), FGF19, and SLC4A2 (encoding AE2)) are associated with ICP. Mutations in one or more of these genes may lead to excessive BA retention in the liver or excretion directly into the blood.^68,71^ For example, MDR3 is an important transporter protein in the transport of BAs from the bile ducts to the gallbladder, and some ICP patients have detected missense mutation or mononucleotide deletion in this gene, resulting in reduced amounts of functional MDR3 protein or loss of activity due to shortening of the protein.^72,73^ (ii) Pregnancy-related hormone levels. In ICP, an increase in the levels of the reproductive hormones estrogen and progesterone leads to cholestasis, which is one of the reasons why ICP often occurs in the second half of pregnancy. Estrogen can internalize BAs transporters, leading to cholestasis.^34^ Progesterone metabolites reduce the export of BAs through the BSEP and competitively inhibit NTCP, thereby reducing the reuptake of BAs by hepatocytes from the portal vein circulation. And, both estrogen and progesterone can desensitize the FXR pathway.^67^

### 2.5 PFIC

PFIC refers to a group of heterogeneous autosomal recessive liver diseases related to the failure of normal hepatocyte formation and bile excretion, leading to cholestasis of hepatocellular origin (Table 1).^36^ PFIC is a group of rare disorders with the true incidence rate precisely unknown. It is now thought to be anywhere between 1/50,000 to 1/100,000 births and nearly 10–15% of children with cholestatic liver disease due to PFIC. Both genders appear to be affected equally.^37^ PFIC has a devastating impact on the lives of children and their families. And if the affected child does not undergo surgery or liver transplantation, few patients survive to the age of 20.^74^ Based on molecular and genetic studies, three types of PFIC (PFIC1, PFIC2, and PFIC3) have been identified and are associated with gene mutations in the hepatocyte transport system involved in bile secretion. Both PFIC1 and PFIC2 are triggered by defects in ATP8B1, which encodes the FIC1 protein, and ABCB11, which encodes the BSEP protein, resulting in impaired bile salt secretion. PFIC3 is formed because of a defect in ABCB4, which encodes the MDR3 protein, eventually impairing biliary tract phospholipid secretion.^38,39^ The main clinical presentation of PFIC is cholestasis, jaundice, and pruritus, with symptoms typically appearing in infancy (for PFIC1 and PFIC2) or early childhood (for PFIC3).^40^ The biochemical features of PFIC1/2 are normal or low levels of GGT, elevated serum BA, and reduced primary BA concentration, while GGT levels are elevated in patients with PFIC3. .^40,75^

The pathogenesis of PFIC differs only slightly between the various types. (i) PFIC1: The product of the ATP8B1 gene, FIC1, is a flippase for phosphatidylserine (PS) that transfers PS from the outer membrane of the hepatic lobular bile duct to the inner membrane, maintaining its asymmetric lipid distribution and thus maintaining the resistance of the bile duct membrane to hydrophobic bile salts, ensuring efficient bile salt transport and preventing cholestasis.^76^ In contrast, mutations in the FIC1 protein lead to disruption of the lipid asymmetric structure, resulting in a reduction in the function of the BA transporter protein, interferes with the secretion of BAs from hepatocytes, leading to cholestasis.^77^ (ii) PFIC2: Loss of BSEP function due to a defect in the ABCB11 gene, leads to reduced bile flow, accumulation of BAs in hepatocytes and hepatocellular damage, which in turn leads to severe hepatocellular cholestasis.^39^ (iii) PFIC3: In patients with PFIC3, MDR3 does not work properly and phosphatidylcholine stops transporting to the bile canaliculi, resulting in abnormal micelle formation, which injures the bile duct epithelium and BECs, leading to cholestasis and destruction of the liver structure by blocking the bile ducts.^78^

### 2.6 PNAC

PNAC is mainly a pediatric disease, and its incidence in infants is much higher than that in adults. It is defined as cholestasis associated with an extended duration of parenteral nutrition (PN) for more than 14 days (Table 1).^41^ It has been reported that the incidence of PNAC in infants receiving PN for at least 2 months may be as high as 50% and the risk of end-stage liver disease in premature infants receiving parenteral nutrition for more than 3 months is as high as 90%.^79^ The expression of PNAC is the same as that in other cholestatic liver diseases, showing elevated concentrations of conjugated bilirubin, serum BA, and transaminase.^41^ Common biochemical criteria include two consecutive measurements of direct bilirubin >2 mg/dL((34.2 μmol/L)) without other causes of liver dysfunction.^42^ Therefore, other causes of cholestasis, such as viral hepatitis, drug-induced changes, and obstruction, must be excluded before an accurate diagnosis can be made.

PNAC is characterized by cholestatic jaundice with or without liver enzyme abnormalities.^80^ Although PN is widely used in clinical practice and is well established to be associated with cholestasis, the exact etiology of PNAC remains unknown. However, PNAC has well-defined risk factors, including infant prematurity, lack of enteral feeding, bacterial overgrowth, repeated infections, and a lack of potential toxicity of the PN components themselves.^41,81^ Several factors have been reported to contribute to the pathogenesis of PNAC. (i) Altered biliary enterohepatic circulation and flora translocation. Under the influence of risk factors for PNAC, the enterohepatic circulation is altered and small intestinal bacterial overgrowth produces lipopolysaccharides (LPS) and peptidoglycan polysaccharide complexes that translocate to the liver through the damaged intestinal mucosa.^82^ Kasmi et al.^83^ used a PNAC mouse model to demonstrate that intestine-derived LPS activates NF-κB via macrophage-derived IL-1β, which interferes with the binding of FXR and liver X receptor (LXR) to the promoters of the tubular bile and sterol transport protein genes (Abcc2, Abcb11, Abcg5/8), respectively, leading to transcriptional repression of the transport proteins and ultimately to cholestasis. (ii) PN itself or specific components of PN may influence hepatic BA transporters^43^ and apoptotic signaling pathways at the genetic level,^44^ thereby promoting the occurrence and development of cholestasis and hepatocyte injury. For example, inappropriate use of carbohydrates and amino acids; carbohydrates in PN preparations mainly refer to glucose. Excess glucose is taken up by the hepatocytes and metabolized to acetyl coenzyme A. Large amounts of acetyl coenzyme A promote adipogenesis, which increases the concentration of triacylglycerol in the liver and promotes the formation of cholestasis. There is evidence that excess amino acids reduce the rate of bile flow, causing BAs accumulation and direct damage to the tubular membrane;^82^ this may be related to several specific amino acids in PN preparations such as tryptophan, methionine and alanine. Animal studies have shown that tryptophan causes an increase in serum glycolic acid levels; methionine affects bile flow which is not dependent on bile salts; and alanine inhibits the uptake of taurocholate.^84^

## 3 Therapeutic strategies

### 3.1 UDCA

UDCA is a kind of medication widely used in a clinical context (Table 2). As a physiological component of BAs, accounting for 1–3% of the total endogenous BAs in the human body, they can dissolve cholesterol gallstones, play an anti-cholestatic role in various cholestatic diseases, and improve liver function.^103^ Therefore, UDCA is increasingly being used to treat cholestatic diseases, including PBC, PSC, DIC, ICP, PFIC, and cystic fibrosis.^2^ The general dose of UDCA is 13–15 mg/kg/D; however, its curative effect remains controversial.^9^

**Table 2.** Therapeutic strategies.

		Mechanism	Dose	Applicable to		references
Existing therapies	UDCA	Reduce bile toxicity; Enhance hepatobiliary secretion; Protect hepatocytes from BA induced apoptosis; Change BA pool	13–15 mg/kg/D	PBC; PSC; ICP; DIC; PFIC; PNAC	^9,27,85,86^	
	SAMe	Form mixed micelles to protect hepatocyte membranes; Increase the fluidity of hepatocyte plasma membrane; Impel the activity of Na+/ K+ ATPase on the membrane; Increase the excretion of hormone metabolites	Intravenous injection: 0.5–1.0 g/D Oral SAMe tablets: 1.0–2.0 g/D	ICP; DIC; PNAC	^68,87–89^	
	FXR agonists (OCA)	Enhances secretion of BAs; Reduce the synthesis and import of BAs; Reduces the time hepatocytes are exposed to potentially toxic BAs	Start with the lowest recommended dose: 5 mg/day, increasing to 10 mg/day after 3 months	PBC	^6,27,90^	
	PPAR agonists(fibrates)	Inhibition of BA synthesis; Stimulate phospholipid excretion; Anti-inflammatory effect (via inhibition of NF-κB signaling)	Bezafibrate (400 mg/D) Fenofibrate (160 mg/D)	PBC	^91–95^	
Novel therapies	FXR agonists (LJN-45291, GS-967492, EDP-30593)	Same as OCA	Still in clinical trials	/	^96–98^	
	FGF-19 analogues (NGM282)	Down regulating BAs synthesis; Prevent recapture of BAs in the ileum; Reducing BAs pool	Still in clinical trials	/	^99,100^	
	PXR agonists	Regulate the expression of genes involved in the detoxification and metabolism of BA	Still in clinical trials	/	^9^	
	TGR5 agonists	Reduce bile acid synthesis; Reduces pro-inflammatory responses; Promotes BECs proliferation and inhibits BA-mediated cell damage	Still in clinical trials	/	^101,102^	

Several potential mechanisms underlying UDCA drug therapy have been identified in clinical and experimental studies. Different types and stages of cholestasis determine the mechanism and effectiveness of UDCA.^85^ In early PBC and PSC, protection of damaged bile duct cells from BAs toxicity may be dominant. Stimulation of toxic secretion from damaged hepatocytes and excretion of bile acids primarily occurs through post-transcriptional mechanisms, involving stimulation of synthesis, targeting, and apical membrane insertion of key transporters.^85^ In ICP and DIC, the promotion of impaired hepatocellular secretion may be key to rapid pruritus relief and improved serum liver function tests.^69,104^ Inhibition of BAs hepatocyte apoptosis can play a role in all cholestatic states characterized by BAs retention in hepatocytes. Therefore, different pathogeneses may facilitate the beneficial role of UDCA in cholestasis induced by various factors. Therefore, the main mechanisms of UDCA are:^86^ (i) protecting bile duct cells from the cytotoxicity of hydrophobic BAs. (ii) stimulation of bile secretion in damaged hepatobiliary cells. (iii) protection of hepatocytes from BA-induced apoptosis at the mitochondrial level.

### 3.2 S-adenosine-L-methionine

S-adenosine-l-methionine (SAMe, also known as SAM or AdoMet) is mainly synthesized and metabolized in vivo by the liver via multiple pathways (Table 2).^105^ As a donor or enzyme inducer, it participates in three important metabolic processes in tissues: transmethylation, transsulfuration, and polyamine biosynthesis.^106^ Exogenous SAMe is likely to be rapidly metabolized, thereby improving overall hepatic metabolic homeostasis.^107^ SAMe may improve cholestasis through several mechanisms. Mixed micelles can be formed to protect hepatocyte membranes from BAs.^68^ In addition, SAMe can increase the fluidity of the hepatocyte plasma membrane, impair the activity of Na+/K+ ATPase on the membrane, and amplify the uptake of bile salts from blood sinuses, thereby reducing intrahepatic cholestasis and preventing hepatocyte injury and necrosis.^87^ Although it has been proposed to improve cholestasis, research on its efficacy is mixed.

Currently, SAMe is mainly used for hepatocyte cholestasis, ICP, and DIC. In a meta-analysis of 102 clinical trials of SAMe by the Agency for Healthcare Research and Quality, it was shown to be efficacious in relieving itching and lowering elevated serum bilirubin levels related to ICP.^108^ In DIC, SAMe has been summarized as an effective treatment. It can relieve pruritus and normalize serum total bilirubin and alkaline phosphatase levels.^109^ The use of SAMe is as follows: initial treatment, intravenous injection of 0.5–1.0 g/d for 2 weeks; maintenance treatment, oral SAMe tablets of 1.0–2.0 g/d.^88,89^

### 3.3 FXR agonists

FXR is a BA receptor with multiple functions, such as regulating the absorption, metabolism, transport, and excretion of BAs (Table 2). FXR is closely related to the development of cholestasis in a clinical context and is a key therapeutic target for cholestasis and other liver diseases. OCA is a semi-synthetic 6-ethyl derivative of chenodeoxycholic acid (CDCA), which acts as an FXR ligand that activates FXR more potently by 100-fold than the natural analog of BA.^110^ In the liver, FXR is expressed at high levels and can directly regulate the expression of several BA transporters, including BSEP, to enhance the secretion of conjugated BAs, improve the clearance of conjugated BAs, reduce of the time of hepatocyte exposure to potentially toxic BAs,^90^ and considerably improve biochemical indices such as ALP and GGT.^28^

Studies^111^ have shown that in vivo administration of OCA can play a beneficial role through the increase of the expression of small heterodimer partner (SHP), BSEP, and MRP2 in the liver, and reduction of the expression of cholesterol 7 α-hydroxylase (CYP7A1), sterol 12α -hydroxylase (CYP8B1), and NTCP mRNA. In clinical studies, OCA has also been demonstrated to have anti-cholestatic, anti-inflammatory, and anti-fibrotic effects in clinical studies, where treatment with 5–10 mg of OCA was found to reduce serum ALP in patients with PSC.^112^ However, OCA treatment is often accompanied by pruritus in a dose-dependent manner. Generally, the lower the initial dose, the better the control of side effects. Therefore, if the drug is well tolerated, side effects such as pruritus during treatment can be alleviated by starting OCA at the lowest recommended dose of 5 mg/day and increasing it to 10 mg/day after three months.^6^ Apart from OCA, other FXR agonists (LJN-452^101^, GS-9674^102^, EDP-305^103^) are emerging as potential therapeutics for cholestasis. They are currently in clinical trials with NCT numbers NCT02516605, NCT02943447 and NCT03394924, all of which are non-bile acid formulations and are therefore predicted to reduce itching and hyperlipidemia compared to OCA.^6^

### 3.4 FGF-19 analogues

Elevated plasma levels of FGF-19 have been found in patients with extrahepatic cholestasis,^113^ suggesting that FGF-19 regulation may provide promising therapeutic potential in such metabolic diseases. Although natural FGF-19 has strong anti-cholestasis and anti-fibrosis activities, it retains its unique tumor-promoting effect.^114^ NGM282, which completely retains the ability to regulate BAs without pro-tumor activity, is indicated as a new therapeutic strategy for cholestasis (Table 2).^115^

In a mouse model of obstructive extrahepatic and intrahepatic cholestasis, NGM282 administration inhibited hepatic CYP7A1 expression, thus reducing the concentration of hepatic BAs and serum liver enzymes and preventing cholestatic liver injury.^99^ In a mouse model of PFIC, NGM282 alleviated liver injury, inflammation, and fibrosis, downregulated the expression of CYP7A1, and reduced BA pool size.^100^ These results indicate that NGM282 is a promising therapeutic agent for cholestasis. In a randomized trial of healthy human volunteers, NGM282 was found to be well tolerated for one week (3 mg/day) and reduced CYP7A1 activity and postprandial serum BA levels, suggesting that NGM282 can provide an effective solution for the treatment of human cholestasis.^116^ In a multicenter phase-two trial in adults with PSC, NGM282 effectively suppressed BA synthesis and downgraded fibrosis markers in patients with PSC.^117^ However, in the context of the design, overall findings, and limitations of the study, it is too early to speculate whether NGM282 has real clinical benefits in PSC and PBC; however, it is undeniable that these results will present the opportunity for further research.

### 3.5 Peroxisome proliferator-activated receptors (PPAR) agonists: Fibrates

The fibrates (bezafibrate and fenofibrate) have also shown beneficial effects as agonists of PPAR in cholestasis. PPARs are a group of nuclear receptors, including PPARα, PPARγ, and PPARδ (also known as PPARß), which are of great interest for the treatment of cholestasis due to their expression in different hepatic parenchymal and non-hepatic parenchymal cell compartments.^15^ Bezafibrate has a similar affinity for the three isomers mentioned above, while fenofibrate is a PPARα-specific agonist.^118^ Studies suggest that the beneficial effects of fibrates in cholestasis appear to be through (i) inhibition of BA synthesis: reduction of CYP7A1 and CYP27A1 mRNA, together with their respective hydroxylase activities, induces promoter activity of the ASBT gene in Caco2 cells.^91^ (ii) Stimulation of phospholipid excretion counteracts the toxic damage of BAs on the bile ducts: fibrates activate PPARα to directly upregulate the expression of human MDR3 (major determinants of biliary phosphatidylcholine secretion), by binding to a specific PPRE located at the gene promoter.^92^ (iii) Anti-inflammatory effect (via inhibition of NF-κB signaling). Fibrates activation of PPARα induces IκBα protein expression in a PPARα-dependent manner, thereby reducing p65-mediated gene activation of the pro-inflammatory cytokine IL-1β.^93^ A number of clinical trials have also validated the beneficial effects of fibrates. A 24-month randomized double-blind placebo-controlled phase 3 clinical trial^94^ reported that PBC patients with an inadequate response to UDCA were given 400 mg of bezafibrate or placebo daily in addition to continued treatment with UDCA. In comparison to the placebo group, patients in the bezafibrate group showed sustained rapid decreases in alkaline phosphatase and bilirubin, reduced liver stiffness and improved liver fibrosis scores. Fenofibrate (160 mg/day) in combination with UDCA for 48 weeks in patients with PBC.^95^

### 3.6 Other targets

Potential treatments for cholestasis also include two other agonists, Pregnane X receptor (PXR) agonists, and G protein receptor 5 (TGR5) agonists. PXR plays an important role in regulating the expression of genes that participate in the detoxification and metabolism of BA, drugs and other toxins, and regulates the expression of CYP3A4 and CYP7A1 SULT2A1, UGT1A1, MDR1/2/3 and organic solute transportersβ (OSTβ).^9^ PXR agonists include LCA and a variety of drugs including rifampicin, statins, corticosteroids, phenobarbital. Among them is the antibiotic rifampicin, a potent human PXR activator that induces upregulation of UGT1A1 and MRP2, promotes bilirubin elimination, increases CYP3A4 expression, and promotes BA detoxification.^119^ The combination of UDCA and rifampicin has been reported to play a possible synergistic beneficial role in patients with non-obstructive cholestasis.^120^ The steroid drug budesonide was shown in an in vitro study to stimulate the apical chloride/bicarbonate exchanger (AE2) in combination with UDCA, producing bicarbonate-rich choleresis and enhancing the bicarbonate umbrella.^121^ Further validated in a phase III clinical trial (NCT00746486).

The broad and beneficial activity of TGR5 makes it a potential therapeutic target, and INT-777 and INT-767, for example, are two agonists of TGR5 that correct bile secretion and cell survival in patients with PBC.^27,122^ And INT-767 has been pointed out to reduce BAs synthesis, increase bicarbonate excretion and down-regulate NF-κB signaling.^101^

## 4 The triangular relationship of gut microbiota-BAs-cholestasis

### 4.1 Gut microbiota in cholestasis

Human beings are estimated to be parasitized by approximately 100 trillion microbial cells, most of which exist in the gut where they form a complex microbial community called the intestinal microbiome.^123^ The gut microbiome is essential for human health and is involved in multiple physiological processes and functions.^124^ Generally speaking, microbiome-host interaction occurs mainly through three mechanisms:^125^ (i) the immunobiome, including the sophisticated interaction between the intestinal microbiota and immune system of the host, balancing immune tolerance to symbiotic bacteria and preventing pathogens and immune response disorders; (ii) the endobiome, where gut microbiota contribute to host physiology by producing numerous metabolites (such as vitamins, secondary BAs, neurotransmitters, etc.). These metabolites function as signaling molecules in the host and serve as substrates for metabolic reactions; (iii) the xenobiome, where the gut microbiome transforms foreign compounds, including nutrients, drugs, and various undefined environmental exposures, leading to the formation of a wide range of metabolites that can be detected in the host blood. Some articles^126–128^ have shown that the pathogenesis of cholestasis is inextricably linked to the ecological imbalance of gut microbiota; however, the potential mechanism is not completely understood. Physically, the bile ducts connect the liver to the gut, and the portal vein transports blood containing the products of intestinal flora to the liver, which then enters the systemic circulatory system. This implies that the liver plays a pivotal role in the interaction between the gut microbiome and host.^125^ Microbiota-derived molecules can activate the immune system and provide assurance that the liver can tolerate beneficial and harmless molecules and metabolites,^129^ serving as a strong line of defense against pathogens and harmful microbial-derived molecules.^130^ Therefore, gut microbiome disorders may be key factors in cholestasis, which is also attributed to the fact that the intestinal microbiome is the core of BA homeostasis.

Tang et al.^127^ comparatively analyzed the gut microbiome in PBC and healthy controls and observed a dramatic reduction in intraindividual flora diversity in PBC (p = 0.03). At the genus level, a decrease in abundance was found in four genera (*Faecalibacterium, Bacteroides, Sutterella*, and *Oscillospira* spp.), and an increase in the abundance of eight genera (*Haemophilus, Veillonella, Clostridium, Lactobacillus, Streptococcus, Pseudomonas, Klebsiella*, and an unknown genus in the family *Enterobacteriaceae* was found to be strongly associated with PBC. Interestingly, UDCA treatment reversed the richness of the six PBC-associated genera. It has also been found that compared with the healthy population, the intestinal bacterial diversity in individuals with PSC is reduced, bacterial richness and evenness are reduced, and some bacterial genera are enriched in the intestines of patients with PSC, including *Veillonella, Enterococcus, Fusobacterium, Lactobacillus, and Streptococcus*.^131^ There are inconsistent findings on the changes in *Clostridium*,^132^ which may be due to the different environment of the host body itself and other pathological states, but it cannot be denied that the intestinal microbiome composition of patients with PSC is altered. This was also evident in PBC, where both cases showed enrichment for specific taxa, such as *Streptococcus, Lactobacillus*, and *Veillonella*. The gut microbiota were also altered in ICP and PNAC. In one study,^133^ researchers identified seven genera (*Escherichia, Shigella, Parabacteroides, Flavonifractor, Atopobium, Turicibacter, Lactobacillus*, and *Megamonas*) whose abundance differed between patients with ICP and healthy individuals. Additionally, there were differences in the family Lactobacillaceae and phylum Proteobacteria between the two groups. In contrast, UDCA treatment modulated the changes in the intestinal microbiota and upregulated the percentage of Bacteroidetes.^134^ Another study^135^ found five genera with statistically significant differences in abundance between PNAC and non-PNAC samples. PNAC was closely related to elevated levels of the gram-negative bacteria *Klebsiella, Veillonella, Enterobacter*, and *Enterococcus* and reduced levels of *Escherichia/Shigella*. Among them, the median relative abundance of *Klebsiella, Enterococcus*, and *Veillonella* was the highest, and the content was higher in PNAC samples.

Overall, the gut microbiome is altered in patients with cholestatic liver disease. There is growing evidence in favor of the gut microbiota can modulate cholestasis. On the premise that the abundance of *Prevotella copri* in the gut of PSC patients is known to be significantly reduced,^136,137^ Jiang et al.^138^ established a PSC mouse model by feeding a diet containing 0.1% 3,5-diethoxycarbonyl-1,4-dihydrocollidine (DDC) and demonstrated that intervention with *P. copri* significantly improved cholestasis. *P. copri* intervention significantly improved cholestasis, and this effect was achieved by affecting the structure of the gut microbiota and its relationship with BAs (the relationship between microbes and bile acids is described in detail later). Administration of *P. copri* may regulate the activity of BA receptors (mainly FXR) by enriching the number of *Muribagulaceae* (family S24–7) and *Clostridium*, thereby improving BAs transport and inhibiting BAs synthesis to alleviate cholestasis in the liver. Oriol Juanola et al.^139^ induced acute cholestasis in germ-free (GF) and altered Schaedler’s flora (ASF) colonized mice by BDL and found that the lack of gut microbiota was related to the obvious aggravation of hepatic cholestasis after BDL. Compared with ASF colonized mice, in the presence of microbiota, ductular reactions, cell proliferation, deposition of collagen 1 and autophagy were all enhanced, while GF mice are more prone to liver inflammation, which is manifested as increased gene expression levels of osteopontin, interleukin (IL)-1β and activation of the ERK/MAPK pathway. The results suggest that this protective effect of microorganisms is relevant to different hepatic gene expression profiles, most of which are associated with tissue repair, metabolic and immune function. Another population-based trial^140^ pointed out that the differential microbial composition of patients with cholestasis also contributes to their unique symbiotic network. For example, a positive correlation between *Ruminococcus* and Bacteroides was found in patients. Since *Ruminococcus* can produce UDCA to alleviate cholestasis,^141^ a further reduction in *Ruminococcus* numbers in the patient’s gut, which may make cholestasis worse. These findings highlight that differences caused by microbiota may influence the process of cholestasis, disease progression may further impair the gut microbiota, which in turn may exacerbate cholestasis, creating a vicious cycle. The positive effects of intestinal flora on host health and the early stages of cholestasis are undeniable and provide the idea and theoretical basis for new therapies based on regulation of the gut microorganisms. However, disease progression may further impair the gut microbiota, which in turn may exacerbate cholestasis, creating a vicious cycle.

### 4.2 Enterohepatic circulation and receptors in cholestasis

**Figure 2.**
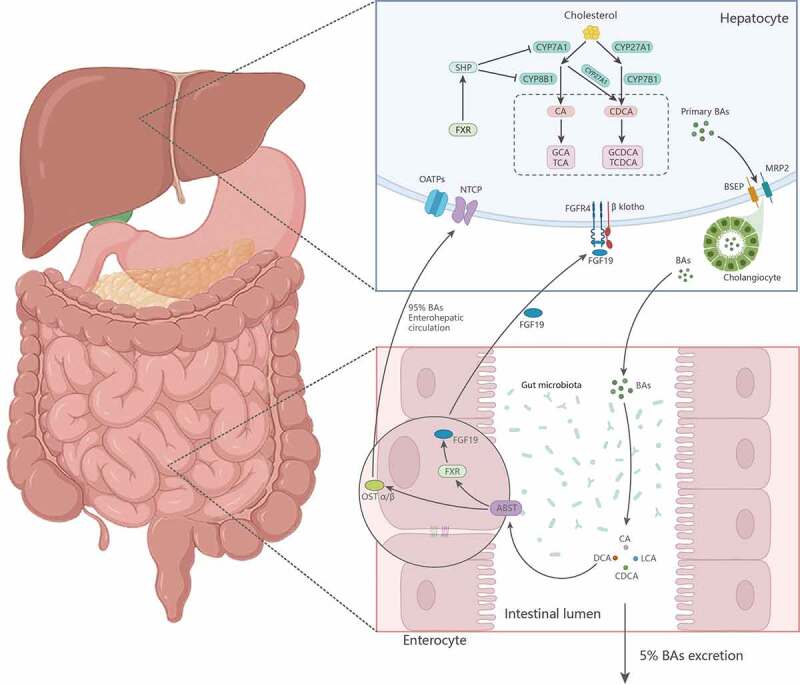
Enterohepatic circulation.

With the expansion of research on the molecular mechanisms of cholestasis, the enterohepatic circulation of BAs has gradually been recognized to play an important role in different types of cholestasis. Reducing enterohepatic circulation can decrease the aggregation of BAs in the liver and circulatory system and reduce the BA pool, which may be an effective means to treat a variety of cholestasis-related diseases. There are two synthetic pathways for BAs in hepatocytes: classical and alternative.^142^ The classical pathway is initiated by the rate-limiting enzyme CYP7A1, which catalyzes the conversion of cholesterol to 7α-hydroxycholesterol. 7α-hydroxycholesterol is then modified by CYP8B1 to produce cholic acid (CA) and CDCA, the two major BAs synthesized in the human liver.^142,143^ BA is synthesized by alternative pathways through sterol 27α-hydroxylase (CYP27A1) and 25-hydroxycholesterol 7α-hydroxylase (CYP7B1).^144^ In the liver, most primary BAs can bind to glycine or taurine to produce conjugated hydrophilic BAs. These BAs are transported from the liver cells to the hepatic duct by BSEP distributed on the cell membrane, through the biliary system, and finally released into the intestine. Small amounts are secreted into the distal ileum or colon, where they can be further modified by intestinal flora and related enzymes into BAs with more hydrophobic abilities, including deoxycholic acid (DCA) and LCA.^145^ Most BAs are reabsorbed into the ileal epithelial cells mainly with the assistance of an apical sodium-dependent BA transporter (ASBT/SLC10A2), and then leave the intestine through OSTα/β located in the basement membrane of the cells and reach the portal system, the liver mediates the reabsorption of BA through NTCP and OATPs.^15,146^ The entire process is known as the enterohepatic cycle, which occurs approximately 4–12 times a day in humans (Figure 2). Approximately 95% of bile-duct excreted BAs are reabsorbed from the ileum or colon, and 5% of BAs are excreted in feces and replenished by de novo synthesis of BAs by hepatocytes.^144,147^

BAs are regulated by a variety of nuclear and membrane receptors, FXR and TGR5 are the main representatives of nuclear receptor and membrane receptor, respectively.^148^ The FXR receptor is highly expressed in hepatocytes and intestines and is a BA sensor that regulates BAs absorption, metabolism, and transport. Therefore, FXR is an important nuclear receptor for maintaining BA enterohepatic circulation and homeostasis, and plays an important role in cholestasis. In cholestasis, FXR can be activated by the BAs of different structures and mediate BAs synthesis and secretion. The most potent FXR ligand was CDCA, followed by CDCA> DCA> CA> LCA.^149^ Modica et al. showed that the activation of FXR receptors reduces intrahepatic BAs thereby alleviating liver injury caused by cholestasis.^150^ FXR activation upregulates SHP expression in the liver and fibroblast growth factors (FGF) 15 (mouse) and 19 (human) in the gut, both of which can reduce hepatic CYP7A1 and CYP8B1 enzyme activity, thereby reducing BA synthesis. Additionally, activation of FXR can also provide an excretion pathway for BA, which is achieved by increasing the expression of canalicular transporters such as BSEP.^148^ Overall, FXR regulates BAs based on enterohepatic circulation mainly through three major pathways: the SHP, FGF15/ FGF19, and c-Jun N-terminal kinase (JNK) pathways.^102,151^ As a principal intracellular BA receptor and critical regulator of BA homeostasis, FXR has emerged as an attractive therapeutic target for the treatment of cholestasis.^152^ Disruption of FXR or the combined absence of FXR and SHP triggers different degrees of cholestasis, whereas activation of FXR upregulates the transcription of BA detoxification enzymes and hepatobiliary transport proteins, facilitates BA clearance and inhibits enzymes associated with BA synthesis, which reduces hepatic BA load and mitigating the toxic cellular damage caused by BA in cholestasis.^153,154^ In cholestasis, FXR not only acts as the regulator of BA, regulating the synthesis, transport, reabsorption and metabolic balance of BA, but also regulates the intestinal microbiota, which is mediated by the antibacterial peptide regulated by FXR.^15^ However, the relationship between intestinal microbiota and cholestasis is not simply one-way, but highly intertwined, which will be introduced in more detail in the following content.

TGR5 is a BA receptor widely present in humans and animals. It is ubiquitously expressed in Kupffer cells, BECs, and liver sinusoidal endothelial cells, and can be activated by LCA, DCA, CDCA, and other BAs.^155^ It can activate various intracellular signaling pathways upon interaction with BAs.^156^ TGR5 contributes to the integrity of the tight junctions between BEC cells and protects BECs from BA toxicity, which explains the markedly reduced survival of TGR5-deficient BECs in response to BA toxicity.^157^ TGR5 activation also promotes BECs proliferation, elevated BA concentrations can reportedly induce the activation of TGR5 in BECs, promote the proliferation of bile duct cells, and inhibit BA-mediated cell damage.^102^ Based on the pro-proliferative and anti-apoptotic properties of TGR5 and its prominent expression in BECs, as well as its extensive involvement in bile secretion and cytoprotection, TGR5 is thought to be involved in the regulation of various diseases caused by cholestasis^158^ and may act as a protector in the process of cholestasis, affecting the development of cholestasis through the regulation of NF-κB, AKT and other related signaling pathways. It has been shown that TGR5 knockdown exacerbates liver injury in models of BDL or LCA-induced cholestasis,^159,160^ and that in the absence of TGR5, cholestasis results in reduced BEC proliferation and increased intrahepatic and extrahepatic BEC damage, which may also lead to the development or progression of PBC, PSC, as the reduction in TGR5-dependent cytoprotective mechanisms (e.g. bicarbonate secretion) makes BECs more susceptible to bile salt toxicity.^161^ Activation of TGR5 triggers the formation of bicarbonate umbrella, promotes the integrity of tight junctions and mediates bile duct bicarbonate secretion via anion exchanger 2, regulates intracellular acid-base balance and protects BECs from BA toxicity.^162^ Further studies^163,164^ have shown that TGR5 activation reduces reactive oxygen species production by inhibiting NF-κB-dependent pro-inflammatory cytokine production and activating Nrf2/HO-1 signaling, and also promotes β-catenin signaling to activate the PI3K/ AKT signaling pathway, thereby inhibiting the inflammatory response to counteract cholestasis. In general, during cholestasis, activation of TGR5 in BECs facilitates the secretion of chloride and bicarbonate, triggers cell proliferation and protects against apoptotic cell death; activation of TGR5 in immune cells suppresses cytokine expression and secretion, thereby reducing systemic as well as hepatic and intestinal inflammation.^165^

### 4.3 Interactions between gut microbiota and BAs

#### 4.3.1 Gut microbiota regulates BAs

##### 4.3.1.1 Biotransformation of BAs by gut microbiota

As mentioned earlier, BAs are synthesized in the liver and released into the gut via the biliary system, and partly into the distal ileum or colon where they are further metabolized by the gut flora. The gut microbes can alter the BA profile as they have different BA metabolizing enzymes. The gut microbiota modulates the chemical diversity of BAs through deconjugation, 7ɑ-dehydroxylation, isomerization, oxidation, desulfurization, and esterification, which subsequently affect their microbial toxicity and intestinal absorption.^166^ The deconjugation and 7ɑ-dehydroxylation are two notable reactions.^147^

Microbial deconjugation, primarily by bile salt hydrolases (BSHs), is a critical first step in further modification of BAs by microbes in the gut environment. BSH-encoding genes have been detected and characterized in various gut microbes, including *Bifidobacterium, Lactobacillus, Enterococcus, Clostridium, Bacteroides* and more.^167,168^ It has been shown that an increase in the abundance of BSH-secreting Bacteroides during pregnancy promotes BA deconjugation, thereby reducing enterocyte BA uptake. BSH-deconjugated BAs are subsequently dehydrated by 7ɑ-dehydroxylation produced by the intestinal flora and are ultimately converted to secondary and tertiary BAs.^169^ However, 7α-dehydroxylation is performed by only a few anaerobic species, and current estimates suggest that only approximately 0.0001% of colonic bacteria can perform this reaction, representing less than 0.025% of the total gut microbiome.^170,171^ 16S rRNA sequence analysis showed that these bacteria were mainly *Clostridium* species, including *C. hiranonis, C. scindens, C. hylemonae* (cluster XIVa), and *C. sordelli* (cluster XI), all of which belong to the phylum *Firmicutes*.^172^ In the large intestine, *Clostridium* species can convert CA and CDCA to DCA and LCA, respectively, via 7α-dehydroxylation.^173^ Oxidation and epoxidation have also received special attention in the microbial modification of BAs and regulation of their metabolic reactions, with some gut microbes synthesizing hydroxysteroid dehydrogenases (HSDHs) capable of reversible redox reactions and hydroxyl epoxidation. HSDHs activity exists in four major phyla of the gut microbiota: *Actinobacteria, Proteobacteria, Firmicutes*, and *Bacteroidetes*. These oxidation reactions can eventually lead to epimerization. However, owing to the lack of appropriate analytical methods, the mechanisms by which microorganisms regulate BA isomerization reactions via HSDHs are poorly understood, but the hydrophobicity and toxicity of these reactive BAs are irrefutable.^147,174^ Disruption of the intestinal microbiota can cause disturbances in BA metabolism, as supported by a study conducted in 2019.^175^ This study found that taurine-conjugated BAs were significantly increased in the plasma and feces of rats treated with various antibiotics, while free BAs were decreased, and the diversity of BAs was also significantly decreased. An earlier study^176^ also noted that gut microbiota had a significant effect on the BA pool. The diversity of the BA pool of germ-free mice was reduced and lacked unbound and secondary BAs. Compared to normal mice, the levels of BAs decreased in the cecum, colon, and feces, but increased in the gallbladder and small intestine. That the microbiota affects FXR signaling was further demonstrated in the experiments of Li et al.^177^ When mice were treated with tempol, tempol decreased Lactobacillus spp. and its BSH activity to alter the intestinal microbiome, leading to a decrease in the Firmicutes: Bacteroides ratio, a decrease in secondary BAs, and accumulation of tauro-β-muricholic acid (T-β-MCA). While secondary BAs are effective FXR agonists and T-β-MCA is FXR antagonists, inhibition of FXR signaling leads to increased BAs synthesis and an expansion of BA pool size, but a decrease in BA diversity due to the decrease of *Clostridium clusters XI* and *XVIa* involved in BA dehydrogenation. This process can also be reversed by *Desulfovibrionales*, which are responsible for the metabolism of sulfur-containing compounds from dietary and host sources.^178^ Taurine is one of the sources of sulfur-containing substances, and H_2_S derived from *Desulfovibrionales* is beneficial to induce the activation of cecal bacteria 7α-dehydroxylase and promote the growth of 7α-dehydroxylated bacteria, so the gut microbiota rich in *Desulfovibrionales* can regulate BA metabolism, so that the intestine produces more secondary BAs.^179^

##### 4.3.1.2 Biosynthesis of BAs by gut microbiota

The regulation of BAs formation by gut microbiota is complex, including several reaction steps catalyzed by at least 17 different enzymes.^180^ However, more importantly, the metabolism of BAs by the gut microbiota can influence the expression of a number of key enzymes involved in the de novo synthesis of BAs,^181^ including CYP7A1, CYP7B1, CYP8B1 and CYP27A1, such as *Clostridium spp*. and *Eubacterium spp*. in *Firmicum* phylum and this process can be postulated to regulate the synthesis of BAs via an FXR-FGF15/19 feedback mechanism. In hepatocytes, FXR regulates BA synthesis via negative feedback from SHP/liver-related homolog-1 (LRH-1)/ LXRα.^181,182^ Once the hepatic FXR is activated, SHP is triggered immediately afterward to repress LRH transcription, and thus CYP7A1 and CYP8B1 transcription. In intestinal cells FXR inhibits BA synthesis via the FXR/FGF19/FGFR4 pathway, where FXR induces FGF19/FGF15, which in turn binds to the FGFR4 and the β-klotho complex, triggering the MAPK/ERK1/2 pathway and ultimately inhibiting the gene expression of CYP7A1 in the liver.^183,184^ Kim et al.^185^ confirmed that the gut cannot produce FXR activators without bacteria. Data from Sama et al.^176^ suggest that the gut microbiota can inhibit CYP7A1 and BA synthesis by reducing T-MCA levels and promoting FXR-dependent FGF15 expression in the ileum, thus suggesting that the gut microbiota regulates the synthesis of BAs through an FXR-FGF15/19 feedback mechanism. And as already mentioned Desulfovibrionales-derived H_2_S can induce hepatic FXR and inhibit CYP7A1 expression and BA synthesis in addition to favoring the growth of 7α-dehydroxylation-containing bacteria.^179^

##### 4.3.1.3 Transportation of BAs by gut microbiota

The microbiota can also regulate BA transport via FXR feedback mechanisms. Activation of FXR in the liver induces BSEP, MRP, OSTα and OSTβ complexes to enhance hepatic elimination of BAs, while reducing BAs reabsorption by inhibiting the downregulation of basolateral NTCP and OATP1B1 and OATP1B3 in hepatocytes by Na^+^/taurocholate.^181^ FXR activation in the intestine upregulates intestinal BA binding protein (I-BABP) to facilitate the passage of bile salts through ileal enterocytes and enhances OSTα and OSTβ expression to aid the passage of BAs from the intestine into the portal circulation.^186^ Also, FXR can regulate the reabsorption of BA by enterocytes and bile duct cells through the SHP and FGF15/19 pathways by down-regulating ASBT.^187^

#### 4.3.2 BAs modulate the gut microbiota structure

##### 4.3.2.1 Direct effects of BAs on gut microbiota

BAs excreted into the intestine are further metabolized by the intestinal microbiota, which, in turn, influences the microbial composition. BAs have been shown to have direct and indirect effects on the intestinal microbiota. Studies have shown that higher BA concentrations exhibit antimicrobial activity with bacterial membranes being their main targets.^188^ When BAs intolerant bacteria are exposed to high concentrations of BAs, BAs will dissolve phospholipids and detach intrinsic membranous proteins, causing the cell membrane to be completely destroyed, resulting in the spilling of intracellular materials.^184,189^ In addition to membrane damage, BAs exhibit direct antimicrobial activity by interfering with the RNA secondary structure, destabilizing macromolecules, causing DNA damage, and promoting protein misfolding, thereby damaging the composition of intestinal microbes.^190^ Cremers et al.^191^ showed that BAs cause extensive protein unfolding and aggregation and that some strains that are defective in reducing oxidative thiol modifications, restoring redox homeostasis, or preventing irreversible protein aggregation under disulfide stress conditions have difficulty surviving in the environment of abnormal BA concentrations. Conversely, BAs can promote the spread of *Bilophila wadsworthia, Escherichia coli*, and *Listeria monocytogenes*.^192,193^ The abundance of Firmicutes was significantly increased in CA-fed rats, and some microorganisms in the *Erysipelotrichi* and *Clostridia*, mainly including the genera *Allobaculum* and *Blautia*, were also increased.^194^ Another study^195^ reported that BA 7α-dehydroxylation bacterial populations collapsed in the absence of BAs. Studies have also ^196,197^ point out that DCA has an extremely potent antimicrobial effect, with 10 times more bactericidal activity than CA, and can severely inhibit the growth of intestinal microflora, including *Lactobacillus, Clostridium perfringens, Bifidobacterium*, and *Bacteroides fragilis*. In general, the reduction of BA pools seems to favor the growth of gram-negative bacteria, which are capable of producing lipopolysaccharides, some of which have pathogenic potential. In contrast, the growth of gram-positive Firmicutes, including some with 7α-dehydroxylation capacity, was observed with an increase in BA pools, thus promoting secondary BA production.^192,197^ Therefore, maintaining bile homeostasis is very important for intestinal microecology

##### 4.3.2.2 Indirect effects of BAs on gut microbiota

Evidence for the bacterial indirect inhibitory effect of BAs can be gathered from mouse models of biliary obstruction that show an over-proliferation of intestinal microbial communities and an increase in bacterial translocation, which, interestingly, can be ameliorated by the use of oral BAs to induce FXR activation, thereby inhibiting bacterial overgrowth.^198^ This is because FXR can induce genes involved in gut protection, including Ang1, iNos and IL18, whose products suppress ileal bacterial overgrowth and alleviate mucosal damage induced by bile duct ligation (BDL).^198^ Mice lacking FXR receptor-expressing cells in the ileum also showed changes in the number of intestinal microorganisms, with an increase and a decrease in the number of *Bacteroidetes* and *Firmicutes* respectively.^199^ The main reason for this was the increased synthesis of BAs after FXR receptor knockdown, suggesting that BAs can counteract the FXR signaling pathway to influence the composition of intestinal microbes. In addition, an in vitro study^200^ assessing the regulation of the antimicrobial peptide cathelicidin expression by CDCA and UDCA found that UDCA and UDCA induced cathelicidin expression via two different nuclear receptors: the FXR and the vitamin D receptor (VDR). Importantly, vitamin D further increased the induction of cathelicidin expression by both bile salts. In turn, the high expression of cathelicidin implies an enhanced defense of the biliary epithelium against microbial invasion.^190^BA can shape the microbiome through FXR-induced antimicrobial peptide production and FXR-induced modulation of host immune responses.

In general, there is a two-way interaction between gut microbiota and BAs. Gut microbiota can regulate the synthesis and metabolism of BAs, and conversely, BAs can alter the composition of the intestinal flora. During cholestasis, the intestinal microbial flora is disturbed, and the destruction of microbial flora further aggravates cholestasis. The diversity of gut microbes is also altered by cholestasis, with the elimination of some beneficial bacteria and the enrichment of other potential pathogens. Interactions between BAs and the gut microbiota are crucial in cholestasis and provide new perspectives for the treatment of this disease.

## 5 Probiotics and cholestasis

### 5.1 Probiotics and BAs

The interaction between microorganisms and BAs has been previously described, and probiotics play an indispensable role in this process. BSH activity is one of the main characteristics of probiotics.^201^ In a large study,^202^ over 300 strains of bacteria were screened and BSH was found to be prevalent among *Bifidobacterium* and *Lactobacillus*, which are two commonly used probiotics. Chiara et al.^203^ demonstrated that the regulation of the intestinal microbiota induced by VSL#3 probiotics (mainly *Lactobacillus* and *Bifidobacterium*) enhanced ileal BAs debinding and promoted fecal BA excretion, and VSL#3 probiotics were found to downregulate hepatic BA synthesis via the intestinal FXR-FGF15 pathway. *Lactobacillus reuteri* NCIMB 30242 and *Lactobacillus casei* YRL577 are both BSH-active probiotics. *L. reuteri* NCIMB 30242 increased circulating unconjugated BA more than two-fold, which can be attributed to increased intestinal BA deconjugation.^204^ *L. casei* YRL577 intervention increased deconjugated BA levels, upregulated FXR and FGF15 mRNA levels, and downregulated ASBT mRNA levels, thereby reducing intestinal BA reabsorption and increasing excretion.^205^ In addition to direct bacterial intervention, the regulation of the gut microbiota through dietary supplementation can also modulate BAs. One study^206^ reported that resveratrol enhanced BA deconjugation and fecal excretion in mice by increasing the abundance of *Lactobacillus* and *Bifidobacterium*, and enhancing BSH activity. In antibiotic-treated mice, resveratrol did not increase hepatic BA synthesis.

### 5.2 The alleviation of cholestasis by probiotics

Cholestasis is often accompanied by an imbalance of intestinal flora, in addition to the accumulation of toxic hepatobiliary acids. Therefore, it is not surprising that modifying intestinal flora, inhibiting BAs synthesis, and promoting BAs excretion are effective ways to alleviate cholestasis. Studies have shown the beneficial effects of certain probiotic species/strains, including *Lactobacillus, Bifidobacterium, and Streptococcus*, on liver disease by the modulating gut microbiota content, improving gut barrier function, reducing bacterial migration, and improving liver damage.^207,208^ Although there are few clinical trials supporting these findings, probiotics have shown potential therapeutic properties for cholestasis.^10^ As already mentioned, probiotics are extensively involved in the BAs anabolic pathways and therefore BAs can be a central target for probiotics to alleviate cholestasis. In this process probiotics can act both directly by modulating FXR receptors and indirectly by regulating the structure of the intestinal flora.

Evidence for direct regulation of FXR by probiotics can be obtained from the experiments of Liu et al. .^17^ Liu tested the preventive effect of probiotic *Lactobacillus rhamnosus GG* (LGG) in cholestatic liver disease using BDL and a multidrug resistance protein 2 knockout (Mdr2−/−) mouse model. The data showed that under the condition of cholestasis, LGG restored hepatic BAs homeostasis by significantly reducing hepatic BAs levels. The beneficial effect of LGG was mediated via the upregulation of the gut FXR-FGF-15 signaling pathway. In another study,^209^ the effect of LGG in preventing cholestasis was determined using a progesterone metabolite, epiallopregnanolone sulfate (PM5S), to mimic an ICP-induced cholestasis mouse model. This study found that LGG may reduce progesterone metabolite-induced BAs dysregulation by activating hepatic FXR and upregulating BSEP. It can be concluded that LGG treatment helps alleviate ICP and cholestatic liver injury, which can be a potential therapeutic strategy for ICP. Chen et al.^16^ further demonstrated the great potential of LGG to alleviate cholestasis in mice model of cholestasis induced by α-naphthylisothiocyanate (ANIT) and clinical antiepileptic drugs valproate acid (VPA).

The indirect effect of probiotics is mainly through the regulation of gut microbiota. Under the acute stimulation of LPS, *L. reuteri SLZX19-12* reduced the proportion of cholestasis-associated microbiome and effectively reduced liver inflammation and hepatocyte apoptosis.^210^ UDCA combined with probiotics can effectively improve liver function in ICP patients, which may be related to the fact that probiotics can regulate gut microbiota, avoid intestinal bacterial overgrowth, and reduce intestinal endotoxemia and BA levels.^211^ The experiment of Liu et al.^17^ mentioned above also shows that LGG treatment can change the intestinal microbiome and enhance gut microbiota that have BSH activity, including *Firmicutes* and *Actinobacteria*. These microorganisms promote BAs deconjugation and reduce hydrophilicity, thereby enhancing BAs excretion. This was further confirmed in the study of hierarchy-assembled dual probiotics system.^16^ It was also found that increased BA excretion by LGG was independent of FXR, as inhibition of FXR did not prevent increased BA excretion by LGG.^17^ It is important to note that despite gut microbiome can balance BAs-based FXR activators and inhibitors by metabolizing BAs, gut flora may produce other kinds of FXR ligands. Probiotics may also enhance FXR activity through direct stimulation of unknown metabolites. Therefore, the selection of FXR ligands from probiotic culture supernatants and intestinal lumen content after probiotic treatment of mice merits further investigation, which will lay the foundation for us to explore more deeply the protective mechanism of probiotics against cholestasis.

In clinical trials, *L. casei* has been shown to alleviate cholestasis. In a randomized controlled trial that examined the influence of probiotics on drug-related liver injury,^10^ the authors showed that an intervention with *L. casei* administered at more than 1 × 10^10^ CFU per day during tuberculosis treatment attenuated abnormal elevations in cholestasis-associated liver indices, which may be associated with the reduction of plasma lipopolysaccharide, repair of intestinal barrier function, and restoration of gut microbiota. At present, a phase 2 trial is evaluating the safety and effectiveness of probiotics in PBC patients with poor UDCA response (NCT03521297). However, probiotics have been rather unsatisfactory in the treatment of cholestasis disorders such as PSC. A randomized placebo-controlled crossover trial of probiotics in patients with PSC revealed that there were no changes in PSC symptoms such as pruritus, fatigue, and stool frequency during the administration of probiotics and no benefit to liver biochemistry or liver function.^212^ Therefore, it is inappropriate to directly deny or affirm the beneficial effects of probiotics. The effects are strain- and dose-specific and may have different effects on cholestasis induced by different factors. Further research is needed to explore the specific molecular mechanisms by which probiotics alleviate cholestasis to explain these phenomena.

**Figure 3.**
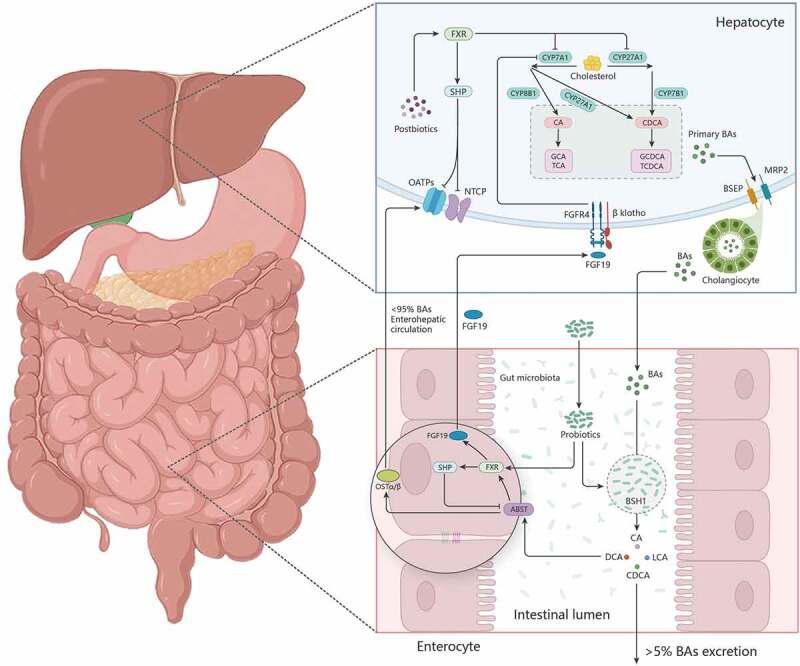
Possible mechanism of probiotics to relieve cholestasis.

According to previous studies, there are three possible mechanisms for probiotics to alleviate cholestasis (Figure 3): (i) probiotics inactivate bacteria, bacterial metabolites, bacterial cell lysates, and other components (i.e., prebiotics) can activate liver FXR to induce the expression of SHP molecules, thus inhibiting the expression of CYP7A1, CYP8B1, and CYP27A1 and reducing the synthesis and accumulation of BAs in the liver. (ii) Probiotics activate intestinal FXR and induce FGF15/FGF19 expression. FGF15/19 arrives in the liver through the portal vein and interacts with the FGF receptor 4 (FGFR4) /βklotho heterodimer complex to induce JNK1/2 and ERK1/2 signaling cascade reactions,^147^ affecting the expression of CYP7A1 and finally regulating BAs anabolism. (iii) Cholestasis can cause disorders in the intestinal microbiome, which can be corrected by probiotics. Probiotics regulate the chemical diversity of BAs by regulating the intestinal microbiome and controlling various metabolic reactions, such as deconjugation, dehydrogenation, dehydroxylation, and isomerization of primary BAs in the gut to produce hydrophobic BAs that are more likely to be excreted in feces.

## 6 Conclusions

Cholestasis is a reduction in bile flow that occurs during a variety of pathologies. Hepatic accumulation of toxic BAs and intestinal microorganism disorders are important factors in cholestatic liver injury; however, the relationship between these three factors is not unidirectional – there is a certain linkage. The main drugs currently available for the treatment of cholestasis include several BAs, BA derivatives, and agonists of receptors, most of which are in the clinical stages of development. Some already-approved medical therapies often fail to achieve the desired effect and sometimes cause side effects. This is mainly because of the complexity of cholestasis. Therefore, despite the progress in research on probiotics in hepatology over the past decade, there are few articles related to their application in cholestasis. As probiotics are known to have the potential to alleviate cholestasis, future research could focus on microbiota-targeted therapies, as well as explore the specific mechanisms by which probiotics alleviate cholestasis and demonstrate their effectiveness in clinical trials or in combination with standard therapies such as UDCA and OCA for cholestasis.

## Data Availability

There are no research data in this paper.
